# Azole-Resistant *Aspergillus fumigatus* Harboring the TR_34_/L98H Mutation: First Report in Portugal in Environmental Samples

**DOI:** 10.3390/microorganisms9010057

**Published:** 2020-12-28

**Authors:** Paulo Gonçalves, Aryse Melo, Marta Dias, Beatriz Almeida, Liliana Aranha Caetano, Cristina Veríssimo, Carla Viegas, Raquel Sabino

**Affiliations:** 1 Department of Infectious Diseases, National Institute of Health Doutor Ricardo Jorge, 1649-016 Lisboa, Portugal; paulo.goncalves@insa.min-saude.pt (P.G.); aryse.melo@insa.min-saude.pt (A.M.); cristina.verissimo@insa.min-saude.pt (C.V.); 2 European Programme for Public Health Microbiology Training (EUPHEM), European Centre for Disease Prevention and Control, 16973 Solna, Sweden; 3 Programa de Pós-Graduação em Microbiologia e Parasitologia, Instituto de Biologia, Universidade Federal de Pelotas, CEP 96010-610 Pelotas, Brazil; 4 H&TRC—Health & Technology Research Center, ESTeSL—Escola Superior de Tecnologia da Saúde, Instituto Politécnico de Lisboa, 1990-096 Lisboa, Portugal; martasfd@gmail.com (M.D.); beatrizltalmeida1@gmail.com (B.A.); liliana.caetano@estesl.ipl.pt (L.A.C.); carla.viegas@estesl.ipl.pt (C.V.); 5 Research Institute for Medicines (iMed.ULisboa), Faculty of Pharmacy, University of Lisboa, 1649-003 Lisboa, Portugal; 6 Public Health Research Centre, NOVA National School of Public Health, Universidade NOVA de Lisboa, 1099-085 Lisboa, Portugal; 7 Comprehensive Health Research Center (CHRC), NOVA Medical School, Universidade NOVA de Lisboa, 1169-056 Lisboa, Portugal; 8 Instituto de Saúde Ambiental, Faculdade de Medicina da Universidade de Lisboa, 1649-028 Lisboa, Portugal

**Keywords:** *Aspergillus fumigatus*, azoles, antifungal resistance, *cyp51A* gene, environment, occupational exposure

## Abstract

Introduction: The frequency in detection of azole-resistant *Aspergillus fumigatus* isolates has increased since 2010. In Portugal, the section *Fumigati* is one of the most frequent, and resistant strains to have been found in clinical and environmental contexts. Although several cryptic species within the *Fumigati* section show intrinsic resistance to azoles, one factor driving (acquired) resistance is selective pressure deriving from the extensive use of azoles. This is particularly problematic in occupational environments where high fungal loads are expected, and where there is an increased risk of human exposure and infection, with impact on treatment success and disease outcome. The mechanisms of resistance are diverse, but mainly associated with mutations in the *cyp51A* gene. Despite TR_34_/L98H being the most frequent mutation described, it has only been detected in clinical specimens in Portugal. Methods: We analyzed 99 *A. fumigatus* isolates from indoor environments (healthcare facilities, spas, one dairy and one waste sorting unit) collected from January 2018 to February 2019 in different regions of Portugal. Isolates were screened for resistance to itraconazole, voriconazole and posaconazole by culture, and resistance was confirmed by broth microdilution. Sequencing of the *cyp51A* gene and its promoter was performed to detect mutations associated with resistance. Results: Overall, 8.1% of isolates were able to grow in the presence of at least one azole, and 3% (isolated from the air in a dairy and from filtering respiratory protective devices in a waste sorting industry) were pan-azole-resistant, bearing the TR_34_/L98H mutation. Conclusion: For the first time in Portugal, we report environmental isolates bearing the TR_34_/L98H mutation, isolated from occupational environments. Environmental surveillance of the emergence of azole-resistant *A. fumigatus* sensu stricto strains is needed, to ensure proper and timely implementation of control policies that may have a positive impact on public and occupational health.

## 1. Introduction

The *Fumigati* section is one of the most prevalent *Aspergillus* sections, in the clinical context as well as in the environment, in Portugal [1,2,3] and elsewhere [4,5,6,7,8,9,10,11]. They can be isolated from air, water, or soil, and can easily contaminate indoor environments. By producing large numbers of small conidia that can become airborne and be inhaled, they can colonize the upper or lower airways, producing mycotoxicosis, allergies, and invasive infections [12,13,14,15,16,17,18,19]. Immunocompromised individuals are at a higher risk of developing disease or aggravate pre-existing respiratory conditions. This fact is particularly important in healthcare settings, where environmental contamination can result in nosocomial outbreaks of fungal respiratory disease [20,21]. Although infection in immunocompetent individuals is not frequent, exposure to largely contaminated environments as it happens in agriculture, in wood and food (particularly animal) industries, and in waste handling increases the risk of infection.

Exposure to bio aerosols in waste handling and sorting plants and in animal farms has been considered as an occupational health problem. These were the main promoters of several respiratory symptoms, namely decline in lung function, asthma, chronic bronchitis, bronchial hyper-responsiveness, wheezing, and coughing. Disposable filtering respiratory protective devices (FRPDs) regularly worn by these workers may constitute a serious occupational hazard, because water vapor and sweat are released, increasing humidity of the material, and together with high temperature, provide favorable conditions for microorganisms’ growth. *Aspergillus fumigatus* has been frequently found on the filters of the FRPD, which can be highlighted as a critical occupational risk [22,23,24].

*Fumigati* cryptic species show intrinsic resistance to several antifungals. However, resistance acquisition in *A. fumigatus* sensu stricto is emerging due to selective pressure caused by the prolonged azole treatment of chronic aspergillosis patients (that can range from several weeks to years, or even a patient’s full lifetime) or due to environmental selective pressure. The mechanisms of azole resistance are often associated with mutations in genes involved in the *A. fumigatus* ergosterol pathway [25], particularly in the *cyp51A* gene which encodes the cytochrome P450 14-α-lanosterol demethylase, the main target of azole antifungals [26]. Point mutations within the *cyp51A* gene (G54, M220) are more frequently associated with prolonged azole prophylaxis/therapy [27,28]. The most common pan-azole resistance mutation is a combination of a 34-bp long tandem repeat in the promoter region and a leucine-to-histidine substitution in codon 98, TR_34_/L98H [25,29,30]. This mutation, firstly described in Dutch *A. fumigatus* isolates, is now spread worldwide [31] due to extensive use of azole fungicides in animal, agricultural, and processing industries.

The selective pressure on *A. fumigatus* is particularly problematic in environments where the use of azoles is a requirement, such as in agriculture, in preservation industries, and sawmills, where an increased probability of emergence of specific occupational health problems has been observed [32,33]. After infection of azole-naïve individuals with these resistant strains, subsequent treatment failure with triazole therapy (the first choice for treatment and prophylaxis of aspergillosis) may occur [34]. Consequently, higher morbidity and mortality rates associated with azole resistance are likely to become a major public health concern [25,29,34,35].

Monitoring the emergence of resistant *A. fumigatus* strains to antifungal drugs, particularly to medical triazoles, becomes essential for the adoption of prevention and control strategies with impact in public health. In Portugal, studies have shown that the frequency of azole-resistant *A. fumigatus* sensu stricto strains is high for itraconazole (ICZ) and posaconazole (PCZ) (up to 92% and 54%, respectively), and lower for voriconazole (VCZ) (up to 3%) [2,3,35]. The TR_34_/L98H mutation has been detected in Portugal in clinical specimens but, to our knowledge, it has not yet been found on environmental isolates [2,3].

The objective of this study was to assess the frequency of cryptic species belonging to *Fumigati* section and determine the frequency of azole resistance in *A. fumigatus* sensu stricto strains isolated from environmental samples. These samples were collected from Portuguese healthcare facilities (hospitals and health centers) and related healthcare environments (thermal spa), and from occupational environments with high fungal loads (waste sorting plants and dairies), where the presence of these fungi may represent a risk for the development of fungal respiratory disease. Our aim was to improve the knowledge of the *Fumigati* epidemiology in these environmental settings and to understand the molecular mechanisms involved in azole resistance in these isolates.

## 2. Materials and Methods

### 2.1. Environmental Sampling

This study was performed using environmental samples collected from January 2018 to February 2019 in different indoor and occupational environments located in several regions of Portugal, with different sampling approaches (Table 1) in the context of enlarged financed studies focusing on occupational exposure to fungi or indoor air quality assessments [22,23,36,37,38,39,40].

Indoor air (50 to 250 L) was collected with a Millipore Air Tester (Millipore, Billerica, MA, USA) using a flow rate of 140 L/min, and impacted directly onto culture media plates, according to the manufacturer’s instructions [39], for the isolation of *A. fumigatus* (section).

Surface samples were collected by swabbing with a 10 cm × 10 cm square stencil, which was disinfected with a 70% alcohol solution between samplings [39]. Fungal contamination was extracted from the swab by washing with 0.9% NaCl with 0.1% Tween80™, for 30 min at 250 rpm on an orbital laboratory shaker (Edmund Bühler SM-30, Hechingen, Germany) [39]. Wash suspensions were inoculated for *A. fumigatus* isolation as described below.

Pieces with 2 cm^2^ (1.4 cm × 1.4 cm) were obtained from filtering respiratory protection devices (FRPDs) and mechanical protection gloves (MPGs) [22,41]. Electrostatic dust collectors (EDC) having a surface exposure area of 0.0209 m (19 × 11 cm) were placed at a minimum 0.93 m above floor level, and dust was allowed to settle on the EDC cloth for 13 to 16 days [37]. Fungal contamination was extracted from the FRPD and MPG pieces, and EDC cloths by washing (as described for surface swabs), and suspensions were inoculated for *A. fumigatus* isolation as described below.

Settled dust samples were weighted, and 1 g of dust was washed with 9.1 mL of 0.9% NaCl with 0.05% Tween80™, for 60 min at 250 rpm [42]. Wash suspensions were inoculated as below.

### 2.2. Isolation of Aspergillus Section Fumigati

**Figure 1:**
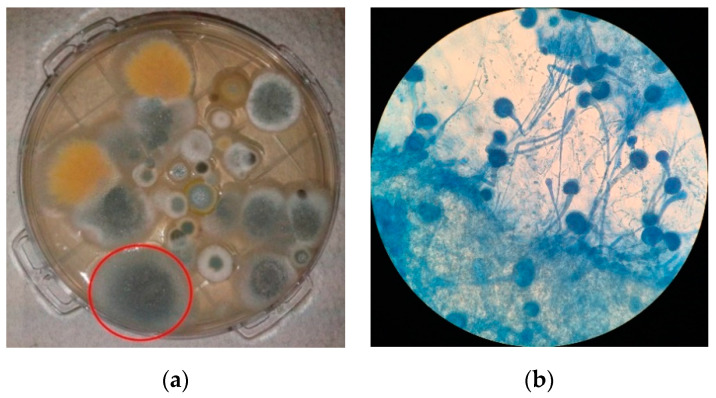
Isolation of *Aspergillus* from air samples: (**a**) Example of a culture media plate resulting from the collection of indoor air by impaction, and from where *Aspergillus* section *Fumigati* (circled red) was isolated; (**b**) Example of a microscopic (400×) observation of a lactophenol blue mount of an *A. fumigatus* isolate.

For *Aspergillus* isolation, 150 µL of the washing suspensions obtained above were inoculated onto malt extract agar (MEA) supplemented with chloramphenicol (0.05%), dichloran–glycerol agar (DG18), Sabouraud dextrose agar (SDA) and SDA supplemented with either 4 mg/L itraconazole (ICZ), 1 mg/L voriconazole (VCZ), or 0.5 mg/L posaconazole (PCZ) [43]. After incubation at 27 °C for 5 to 7 days, fungal species were identified at section level by macroscopic and microscopic morphology using a tease mount or Scotch tape mount and lactophenol cotton blue mount procedures and using identification atlases [44,45] (Figure 1). *Aspergillus fumigatus* isolates were selected for further characterization. These procedures followed the algorithm previously suggested to assess the presence of *A. fumigatus* resistant strains [33].

### 2.3. Molecular Identification of Aspergillus Isolates

*Aspergillus fumigatus* isolates were confirmed by calmodulin [46] or beta-tubulin [47] sequencing. Briefly, amplifications were performed in a 25 µL volume reaction of Illustra PureTaq Read-to-Go PCR beads (GE Healthcare, Buckinghamshire, UK), containing 15 pmol of the primers cmd5/cmd6 or βtub1/βtub2 (for calmodulin or beta-tubulin amplification, respectively) and 4 µL of *Aspergillus* genomic DNA extracted with the High Pure PCR Template Preparation Kit (Roche Diagnostics GmbH, Mannheim, Germany) according to the manufacturer’s instructions. Amplifications were carried with the following thermocycling conditions: 1) for calmodulin an initial denaturation at 95 °C for 10 min, followed by 38 cycles of 95 °C for 30 s, 55 °C for 30 s, and 72 °C for 1 min, and a last final extension step of 72 °C for 7 min; 2) for beta-tubulin an initial denaturation at 94 °C for 2 min, followed by 30 cycles of 94 °C for 30 s, 55 °C for 30 s, and 72 °C for 45 s, and a final extension step of 72 °C for 5 min. PCR products were analyzed by electrophoresis through 2% agarose gels and the resultant PCR amplicons were purified using the ExoSAP-IT enzyme system (USB Corporation, Cleveland, OH, USA), according to the manufacturer’s instructions. Sequencing of the forward strand was performed with the BigDye terminator v. 1.1 Cycle sequencing kit (Applied Biosystems) in the thermal cycler with the following conditions: an initial denaturation at 96 °C for 5 s, followed by 30 cycles of 96 °C for 10 s, 50 °C for 5 s and 60 °C for 4 min, followed by one cycle of 72 °C for 5 min. The resultant nucleotide sequences were edited using the program GeneStudio™ Professional Edition v. 2.2.0.0 and aligned with the program MEGA v. 10.0.5. These sequences were compared with sequences deposited in the GenBank database (Bethesda, MD, USA) in order to achieve the identification to species level.

### 2.4. Screening for Azole-Resistant Isolates

A first characterization of the resistance pattern of the *A. fumigatus* sensu stricto isolates was carried out using screening media made of Sabouraud dextrose agar supplemented with ICZ, VCZ or PCZ [48]. The reference strain *A. fumigatus* ATCC 204305 was used as negative control and the pan-azole-resistant strain TR_34_/L98H (kindly provided by Jacques Meis, Canisius-Wilhelmina Hospital, Nijmegen, The Netherlands) was used as positive control. Strains that were not able to grow in the azole-supplemented plates were considered as susceptible to the respective azoles at tested concentrations, according to EUCAST guidelines [48]. On the other hand, isolates that grew in at least one of the screening media were selected for further analysis.

### 2.5. Antifungal Susceptibility Testing

The M38-A2 protocol from the Clinical and Laboratory Standards Institute (CLSI) was applied for determining the minimal inhibitory concentrations (MIC) for ICZ, VCZ and PCZ [35,49]. The final concentrations of drugs in the wells ranged from 0.0156 to 8 μg/mL. An internal control strain (*A. flavus* ATCC 204304) with known susceptibility was included in each run as a positive control of the antifungals’ potency. Sabouraud dextrose agar plates were inoculated with the final inoculum to check the number of colony-forming units in the inoculum. Plates were incubated at 35 °C and examined after 48 h incubation. Absence of visual growth defined the MIC. Breakpoints for mold testing have not been established by the CLSI (breakpoints for voriconazole and *A. fumigatus* have been set). The CLSI epidemiological cut-off values (ECVs) used were 1 mg/L for ICZ, 1 mg/L for VCZ, and 0.5 mg/L for PCZ [50,51,52]. Isolates with high MICs were tested in triplicate to confirm the obtained results.

### 2.6. Molecular Identification of Resistance Markers

Azole-resistant isolates were tested by a multiplex real-time PCR which screens for TR_34_/L98H and TR46/Y121F/T289A mutations found in the *cyp51A* gene and its promoter (AsperGenius^®^ multiplex real-time PCR assay (PathoNostics, Maastricht, The Netherlands) on the RotorGene Q instrument (Qiagen, Hilden, Germany), following the manufacturer’s instructions), and/or by sequencing of the *cyp51A* gene and its promoter as described [53,54]. Nucleotide sequences were edited and aligned as described above.

## 3. Results

A total of 142 environmental samples were studied (Table 2), the majority of which (114, 80.3%) were obtained from the analyzed waste sorting plant.

*Aspergillus fumigatus* (section) were isolated from all sources studied, particularly from the waste sorting plant (76/114, 66.7%, positive samples) and healthcare units (hospitals/health centers; 19/20, 95.0%, positive samples), for a total of 99/142 (69.7%) isolates. From these, 93/99 (94.0%) were identified as *A. fumigatus* sensu stricto. For the remaining six isolates, identification to species level was not possible because no PCR product for calmodulin or beta-tubulin sequencing could be obtained. Two isolates, obtained from one air sample from a hospital and from an FRPD from a waste sorting worker, were initially classified as *A. fumigatus* based on macroscopic and microscopic characteristics and later identified as *A. sidowii* and *Penicillium* spp., respectively, by calmodulin sequencing and were not considered in the analysis.

Eight out of the 99 *A. fumigatus* sensu stricto isolates (8.1%), obtained from air samples from a diary and a hospital, and from FRPDs worn by workers of a waste sorting plant, grew on at least one of the screening media (Table 3). Three isolates (3.0%) were confirmed as resistant to ICZ, VCZ and PCZ, and sequencing of the *cyp51A* gene and its promoter revealed the TR_34_/L98H mutation in all three isolates. In addition, the N248K mutation was also detected in two isolates. No other mutations were found.

## 4. Discussion

The presence of azole-resistant isolates in Portugal has been reported both in clinical specimens and in the environment [2,3,35,55]. To the best of our knowledge, the TR_34_/L98H mutations, commonly found in the *cyp51A* gene of azole-resistant isolates, were only reported in clinical specimens in this country. Other mutations in the *cyp51A* gene were reported in Portuguese environmental azole-resistant *A. fumigatus* isolates [3], but none undoubtedly described as being associated with resistance. Thus, in this study, we present the first evidence of environmental pan-azole-resistant strains circulating in Portugal harboring the TR_34_/L98H mutations. These resistant strains were isolated from FRPDs worn by waste sorting workers during their working shift and from one air sample from a dairy, which is a strong indicator of the increased risk of disease to which workers from these environments are exposed.

In one of our research group’s publications [56] the literature on occupational and indoor exposure to *Aspergillus* and potential health effects associated with that exposure was reviewed. Among the workplaces with high fungal contamination and potentially high levels of mycotoxins, waste sorting plants stand as one of the environments with the highest fungal contamination [57,58]. This is not only due to the type of materials being processed and the consequent availability of nutrients, but also due to the presence of a high concentration of indoor dust particles, to the deposition of waste indoors, and to the building materials, all of which favor fungal growth and sporulation [56].

*Aspergillus* is also described as one of the most frequent fungi in animal production farms [58,59]. The higher fungal contamination is probably a result of the higher animal density and confined production, conditions that promote the multiplication of microorganisms. Nevertheless, those are not the only sources of contamination, and animal feed and/or animal litter must be considered as potential sources, because their distribution may generate aerosolized particles that remain in the air or are deposited onto the floor for a long time. Cereal-based feed and wood shavings used in bedding can introduce resistant fungal strains into animal production farms. This may happen as a result of the azole pressure exerted in fungal selection, a consequence of the systemic use of azole and azole-based fungicides in the protection of feed crops [60,61] and in wood processing and preservation [32,62]. Although correct management and protection equipment may protect workers in these occupational environments, azole residues are still spread through the environment and can have a negative health impact through their toxicity and persistence in the environment, while boosting the development of azole-resistant fungal strains [63,64].

By working in close contact with these materials, waste sorting and animal production workers are at increased risk of cumulative exposure to fungal particles and mycotoxins, which can reach their respiratory systems and result in occupationally acquired respiratory diseases [56]. Of particular importance, exposure of these workers to azole-resistant isolates may lead to treatment failure, and consequently, significant impact on patient management and associated health costs. Therefore, strategies to prevent or minimize the occupational exposure to *Aspergillus* become of paramount importance and should include, but not be limited to, the correct use, storage and elimination of personal protective equipment, particularly FRPDs, and the implementation of continuous assessment and characterization of the *Aspergillus* burden in such environments.

The obtained results also validate a previously proposed algorithm aiming to support and provide guidance from fieldwork in assessed occupational environments (which sampling methods should be applied) to the bench work (what analysis should be performed) for exposure assessors [33]. Thus, in occupational environments where the azole pressure is high, such as vineyards, sawmills, and waste sorting plants, just to name a few, the frequency of cryptic species and of azole resistance of *A. fumigatus* sensu stricto should be assessed as routine to achieve an accurate risk characterization. In high-load environments, such as in the waste sorting unit and the dairy farm assessed, results are most certainly underestimated; the recovery of the *Aspergillus* section *Fumigati* isolates is a challenging task due to overgrowth of other fungi isolates with fast growth rates, such as Mucorales and *Chrysonilia sitophila* [22,33,37,39,40,42]. Additionally, isolate recovery relies only on the viable component of the fungal contamination. Thus, the culture-based methods drawbacks should be considered [22,33].

Several *Fumigati* isolates from hospital environments were also analyzed in this study. Despite no mutations having been detected, monitoring these environments for the frequency of cryptic species (intrinsically resistant to azoles) and for the emergence of resistant *A. fumigatus* strains to antifungal drugs, particularly to medical triazoles, becomes essential for the adoption of prevention and control strategies in order to reduce the mortality associated with these infections in at risk patients.

Azole-resistant *A. fumigatus* strains will probably become more frequent in the future, as a consequence of natural evolution and by selective pressure due to the use of triazoles in medicine and in the environment, with important public health implications. The widespread use of triazoles, in particular, has become the major driver for the clonal expansion of triazole-resistant *A. fumigatus* genotypes, particularly at local level [65]. Therefore, the continued surveillance of *Aspergillus*, both in the clinical and in the occupational environments, at local and regional levels, is of paramount importance for the control of the emergence of resistance and, ultimately, for the prevention of aspergillosis.

## Figures and Tables

**Table 1:** Indoor and occupational environments assessed and applied sampling approaches.

Type and Number of Indoor/Occupational Environments Assessed	Sampling Approaches
Dairy (*n* = 1)	Air impaction
Hospital (*n* = 2)/Health Centre (*n* = 10)	Air impaction
	EDC
	Settled dust
	Surface swab
Thermal spa (*n* = 5)	Air impaction
	EDC
Waste sorting plant (*n* = 1)	FRPD
	MPG

EDC—Electrostatic dust collector; FRPD—Filtering respiratory protection devices; MPG—Mechanic protection gloves.

**Table 2:** Number of samples screened per sampling approach and type of indoor/occupational environment assessed, and number of samples positive for *Fumigati* section.

Indoor/Occupational Environment	No. of Samples Per Sampling Approach							No. of Samples Positive for *Fumigati* Section (%)
	Air	EDC	Settled Dust	Surface Swab	FRPD	MPG	Total (%)	
Waste sorting plants	0	0	0	0	113	1	114 (80.3)	76 (66.7)
Hospital/Health Centre	13	1	5	1	0	0	20 (14.1)	19 (95.0)
Thermal spa	7	0	0	0	0	0	7 (4.9)	3 (42.9)
Dairy	1	0	0	0	0	0	1 (0.7)	1 (100.0)
Total (%)	21 (14.8)	1 (0.7)	5 (3.5)	1 (0.7)	113 (79.6)	1 (0.7)	142 (100.0)	99 (69.7)

EDC—Electrostatic dust collectors; FRPD—Filtering respiratory protection devices; MPG—Mechanic protection gloves.

**Table 3:** Growth of resistant *A. fumigatus* isolates in different screening media, minimal inhibitory concentrations for ICZ, VCZ and PCZ, and mutations found on the *cyp51A* gene and its promoter.

Isolate Number	Source	Azole Screening Media			Minimal Inhibitory Concentration (mg/L)			*cyp51A* Mutations
		ICZ	VCZ	PCZ	ICZ	VCZ	PCZ	
VA299CP	Dairy air	+	+	+	4	4	2	TR_34_/L98H
VA610CP	Hospital air	±	−	−	2	0.5	0.5	No mutation detected
VA873CP	Waste sorting plant FRPD	+	+	+	4	2	1	TR_34_/L98H
VA978CP	Waste sorting plant FRPD	−	+	−	1	0.25	0.25	No mutation detected
V1207CP	Waste sorting plant FRPD	−	−	+	1	0.5	0.5	No mutation detected
VA1209CP	Waste sorting plant FRPD	+	−	+	8	4	1	TR_34_/L98H
VA1215CP	Waste sorting plant FRPD	−	+	+	1	0.25	0.125	N248K
VA1216CP	Waste sorting plant FRPD	−	+	−	1	0.25	0.25	N248K

ICZ: itraconazole; VCZ: voriconazole; PCZ: posaconazole; −: negative (no growth); ±: residual growth (growth of only one or few small colonies; +: relevant growth (growth similar to positive control).

## Data Availability

The data presented in this study are available on request from the corresponding author. The data are not publicly available due to privacy of sampling locations.

## References

[1] Sabino R., Veríssimo C., Parada H., Brandão J., Viegas C., Carolino E., Clemons K.V., Stevens D.A. (2014). Molecular screening of 246 Portuguese *Aspergillus* isolates among different clinical and environmental sources. Med. Mycol..

[2] Pinto E., Monteiro C., Maia M., Faria M.A., Lopes V., Lameiras C., Pinheiro D. (2018). *Aspergillus* Species and Antifungals Susceptibility in Clinical Setting in the North of Portugal: Cryptic Species and Emerging Azoles Resistance in *A. fumigatus*. Front. Microbiol..

[3] Monteiro C., Pinheiro D., Maia M., Faria M.A., Lameiras C., Pinto E. (2019). *Aspergillus* species collected from environmental air samples in Portugal—Molecular identification, antifungal susceptibility and sequencing of *cyp51A* gene on *A. fumigatus* sensu stricto itraconazole resistant. J. Appl. Microbiol..

[4] Rüping M., Gerlach S., Fischer G., Lass-Florl C., Hellmich M., Vehreschild J., Cornely O.A. (2011). Environmental and clinical epidemiology of *Aspergillus terreus*: Data from a prospective surveillance study. J. Hosp. Infect..

[5] Mobin M., Salmito M.D.A. (2006). Microbiota fúngica dos condicionadores de ar nas unidades de terapia intensiva de Teresina, PI. Rev. Soc. Bras. Med. Trop..

[6] James M.J., Lasker B.A., McNeil M.M., Shelton M., Warnock D.W., Reiss E. (2000). Use of a Repetitive DNA Probe to Type Clinical and Environmental Isolates of *Aspergillus flavus* from a Cluster of Cutaneous Infections in a Neonatal Intensive Care Unit. J. Clin. Microbiol..

[7] Alastruey-Izquierdo A., Mellado E., Cuenca-Estrella M. (2012). Current section and species complex concepts in *Aspergillus*: Recommendations for routine daily practice. Ann. N. Y. Acad. Sci..

[8] Balajee S.A., Kano R., Baddley J.W., Moser S.A., Marr K.A., Alexander B.D., Andes D., Kontoyiannis D.P., Perrone G., Peterson S. (2009). Molecular Identification of *Aspergillus* Species Collected for the Transplant-Associated Infection Surveillance Network. J. Clin. Microbiol..

[9] Krishnan S., Manavathu E.K., Chandrasekar P.H. (2009). *Aspergillus flavus*: An emerging non-fumigatus *Aspergillus* species of significance. Mycoses.

[10] Lortholary O., Gangneux J.-P., Sitbon K., Lebeau B., De Monbrison F., Le Strat Y., Coignard B., Dromer F., Bretagne S. (2011). Epidemiological trends in invasive aspergillosis in France: The SAIF network (2005–2007). Clin. Microbiol. Infect..

[11] Steinbach W.J., Marr K.A., Anaissie E.J., Azie N., Quan S.-P., Meier-Kriesche H.-U., Apewokin S., Horn D.L. (2012). Clinical epidemiology of 960 patients with invasive aspergillosis from the PATH Alliance registry. J. Infect..

[12] Agarwal R., Chakrabarti A., Shah A., Gupta D., Meis J.F., Guleria R., Moss R., Denning D.W. (2013). ABPA complicating asthma ISHAM working group Allergic bronchopulmonary aspergillosis: Review of literature and proposal of new diagnostic and classification criteria. Clin. Exp. Allergy.

[13] Dutre T., Al Dousary S., Zhang N., Bachert C. (2013). Allergic fungal rhinosinusitis—More than a fungal disease?. J. Allergy Clin. Immunol..

[14] Hinojosa M., Fraj J., De La Hoz B., Alcazar R., Sueiro A. (1996). Hypersensitivity pneumonitis in workers exposed to esparto grass (*Stipa tenacissima*) fibers. J. Allergy Clin. Immunol..

[15] Lai H., Mo X., Yang Y., He K., Xiao J., Liu C., Chen J., Lin Y. (2014). Association between aflatoxin B1 occupational airway exposure and risk of hepatocellular carcinoma: A case-control study. Tumor Biol..

[16] Marr K.A., Carter R.A., Boeckh M., Martin P., Corey L. (2002). Invasive aspergillosis in allogeneic stem cell transplant recipients: Changes in epidemiology and risk factors. Blood.

[17] Matsuse H., Tsuchida T., Fukahori S., Kawano T., Nishino T., Fukushima C., Kohno S. (2013). Dissociation between sensitizing and colonizing fungi in patients with allergic bronchopulmonary aspergillosis. Ann. Allergy Asthma Immunol..

[18] Rick E.M., Woolnough K., Pashley C.H., Wardlaw A.J. (2016). Allergic Fungal Airway Disease. J. Investig. Allergol. Clin. Immunol..

[19] Selman M., Pardo A., King T.E. (2012). Hypersensitivity Pneumonitis. Am. J. Respir. Crit. Care Med..

[20] Alangaden G.J. (2011). Nosocomial Fungal Infections: Epidemiology, Infection Control, and Prevention. Infect. Dis. Clin. N. Am..

[21] Godeau C., Reboux G., Scherer E., Laboissiere A., Lechenault-Bergerot C., Millon L., Rocchi S. (2020). Azole-resistant *Aspergillus fumigatus* in the hospital: Surveillance from flower beds to corridors. Am. J. Infect. Control..

[22] Viegas C., Dias M., Almeida B., Caetano L.A., Carolino E., Gomes A.Q., Twarużek M., Kosicki R., Grajewski J., Marchand G. (2020). Are workers from waste sorting industry really protected by wearing Filtering Respiratory Protective Devices? The gap between the myth and reality. Waste Manag..

[23] Viegas C., Twarużek M., Dias M., Almeida B., Carolino E., Soszczyńska E., Ałtyn I., Viegas S., Caetano L.A. (2020). Cytotoxic effect of filtering respiratory protective devices from the waste sorting industry: Is in vitro toxicology useful for risk characterization?. Environ. Res..

[24] Basinas I., Sigsgaard T., Heederik D., Takai H., Omland Ø., Andersen N.T., Wouters I.M., Bønløkke J.H., Kromhout H., Schlünssen V. (2012). Exposure to inhalable dust and endotoxin among Danish livestock farmers: Results from the SUS cohort study. J. Environ. Monit..

[25] Van Der Torre M.H., Novak-Frazer L., Rautemaa-Richardson R. (2020). Detecting Azole-Antifungal Resistance in *Aspergillus fumigatus* by Pyrosequencing. J. Fungi.

[26] Pontes L., Beraquet C.A.G., Arai T., Pigolli G.L., Lyra L., Watanabe A., Moretti M.L., Schreiber A. (2019). *Aspergillus fumigatus* Clinical Isolates Carrying CYP51A with TR34/L98H/S297T/F495I Substitutions Detected after Four-Year Retrospective Azole Resistance Screening in Brazil. Antimicrob. Agents Chemother..

[27] Chen J., Li H., Li R., Bu D., Wan Z. (2005). Mutations in the *cyp51A* gene and susceptibility to itraconazole in *Aspergillus fumigatus* serially isolated from a patient with lung aspergilloma. J. Antimicrob. Chemother..

[28] Escribano P., Recio S., Peláez T., González-Rivera M., Bouza E., Guinea J. (2011). In Vitro Acquisition of Secondary Azole Resistance in *Aspergillus fumigatus* isolates after Prolonged Exposure to Itraconazole: Presence of Heteroresistant Populations. Antimicrob. Agents Chemother..

[29] Pérez-Cantero A., López-Fernández L., Guarro-Artigas J., Capilla J. (2020). Azole resistance mechanisms in *Aspergillus*: Update and recent advances. Int. J. Antimicrob. Agents.

[30] Macedo D., Devoto T.B., Pola S., Finquelievich J.L., Cuestas M.L., Garcia-Effron G. (2020). A Novel Combination of *CYP51A* Mutations Confers Pan-Azole Resistance in *Aspergillus fumigatus*. Antimicrob. Agents Chemother..

[31] Dudakova A., Spiess B., Tangwattanachuleeporn M., Sasse C., Buchheidt D., Weig M., Groß U., Bader O. (2017). Molecular Tools for the Detection and Deduction of Azole Antifungal Drug Resistance Phenotypes in *Aspergillus* Species. Clin. Microbiol. Rev..

[32] Jeanvoine A., Rocchi S., Reboux G., Crini N., Crini G., Millon L. (2017). Azole-resistant *Aspergillus fumigatus* in sawmills of Eastern France. J. Appl. Microbiol..

[33] Viegas C., Almeida B., Caetano L.A., Afanou A., Straumfors A., Veríssimo C., Gonçalves P., Sabino R. (2020). Algorithm to assess the presence of *Aspergillus fumigatus* resistant strains: The case of Norwegian sawmills. Int. J. Environ. Health Res..

[34] European Centre for Disease Prevention and Control (ECDC) (2013). Risk Assessment on the Impact of Environmental Usage of Triazoles on the Development and Spread of Resistance to Medical Triazoles in Aspergillus Species. https://ecdc.europa.eu/sites/portal/files/media/en/publications/Publications/risk-assessment-impact-environmental-usage-of-triazoles-on-Aspergillus-spp-resistance-to-medical-triazoles.pdf.

[35] Sabino R., Carolino E., Veríssimo C., Martinez M., Clemons K.V., Stevens D.A. (2016). Antifungal susceptibility of 175 *Aspergillus* isolates from various clinical and environmental sources. Med. Mycol..

[36] Viegas S., Assunção R., Twaruźek M., Kosicki R., Grajewski J., Viegas C. (2020). Mycotoxins feed contamination in a dairy farm—Potential implications for milk contamination and workers’ exposure in a One Health approach. J. Sci. Food Agric..

[37] Viegas C., Twarużek M., Lourenço R., Dias M., Almeida B., Caetano L.A., Carolino E., Gomes A.Q., Kosicki R., Soszczyńska E. (2020). Bioburden Assessment by Passive Methods on a Clinical Pathology Service in One Central Hospital from Lisbon: What Can it Tell Us Regarding Patients and Staff Exposure?. Atmosphere.

[38] Viegas C., Almeida B., Monteiro A., Paciência I., Rufo J., Aguiar L., Lage B., Gonçalves L.M.D., Caetano L.A., Carolino E. (2020). Exposure assessment in one central hospital: A multi-approach protocol to achieve an accurate risk characterization. Environ. Res..

[39] Viegas C., Almeida B., Monteiro A., Caetano L.A., Carolino E., Gomes A.Q., Twarużek M., Kosicki R., Marchand G., Viegas S. (2019). Bioburden in healthcare centers: Is the compliance with Portuguese legislation enough to prevent and control infection?. Build. Environ..

[40] Viegas C., Dias M., Almeida B., Carolino E., Gomes A.Q., Viegas S. (2020). Aspergillus spp. burden on filtering respiratory protective devices. Is there an occupational health concern?. Air Qual. Atmos. Health.

[41] Viegas C., Twarużek M., Dias M., Almeida B., Carolino E., Kosicki R., Soszczyńska E., Grajewski J., Caetano L.A., Viegas S. (2020). Assessment of the microbial contamination of mechanical protection gloves used on waste sorting industry: A contribution for the risk characterization. Environ. Res..

[42] Viegas C., Almeida B., Monteiro A., Paciência I., Rufo J.C., Carolino E., Quintal-Gomes A., Twarużek M., Kosicki R., Marchand G. (2019). Settled dust assessment in clinical environment: Useful for the evaluation of a wider bioburden spectrum. Int. J. Environ. Health Res..

[43] The European Committee on Antimicrobial Susceptibility Testing (EUCAST) (2018). Breakpoint Tables for Interpretation of MICs for Antifungal Agents. 9.0. https://www.eucast/clinical_breakpoints/.

[44] de Hoog G.S., Guarro J., Gené J., Figueras M.J. (2016). Atlas of Clinical Fungi—The Ultimate Bench Tool for Diagnosis. 4.1.

[45] Campbell C.K., Johnson E.M., Warnock D.W. (1996). Identification of Pathogenic Fungi.

[46] Hong S.-B., Go S.-J., Shin H.-D., Frisvad J.C., Samson R.A. (2005). Polyphasic taxonomy of *Aspergillus fumigatus* and related species. Mycologia.

[47] Staab J.F., Balajee S.A., Marr K.A. (2009). *Aspergillus* Section *Fumigati* Typing by PCR-Restriction Fragment Polymorphism. J. Clin. Microbiol..

[48] Guinea J., Verweij P., Meletiadis J., Mouton J.W., Barchiesi F., Arendrup M.C., Arikan-Akdagli S., Castanheira M., Chryssanthou E., Friberg N. (2019). How to: EUCAST recommendations on the screening procedure E.Def 10.1 for the detection of azole resistance in *Aspergillus fumigatus* isolates using four-well azole-containing agar plates. Clin. Microbiol. Infect..

[49] Clinical and Laboratory Standards Institute (2008). M38-A2 Reference Method for Broth Dilution Antifungal Susceptibility Testing of Filamentous Fungi; Approved Standard.

[50] Clinical and Laboratory Standards Institute (2020). M59 Epidemiological Cutoff Values for Antifungal Susceptibility Testing.

[51] Buil J.B., Hagen F., Chowdhary A., Verweij P.E., Meis J.F. (2018). Itraconazole, Voriconazole, and Posaconazole CLSI MIC Distributions for Wild-Type and Azole-Resistant *Aspergillus fumigatus* Isolates. J. Fungi.

[52] Espinel-Ingroff A., Diekema D.J., Fothergill A., Johnson E., Pelaez T., Pfaller M.A., Rinaldi M.G., Canton E., Turnidge J. (2010). Wild-Type MIC Distributions and Epidemiological Cutoff Values for the Triazoles and Six *Aspergillus* spp. for the CLSI Broth Microdilution Method (M38-A2 Document). J. Clin. Microbiol..

[53] Prigitano A., Venier V., Cogliati M., De Lorenzis G., Esposto M.C., Tortorano A.M. (2014). Azole-resistant *Aspergillus fumigatus* in the environment of northern Italy, May 2011 to June 2012. Eurosurveillance.

[54] Mellado E., Diaz-Guerra T.M., Cuenca-Estrella M., Rodriguez-Tudela J.L. (2001). Identification of Two Different 14-α Sterol Demethylase-Related Genes (*cyp51A* and *cyp51B*) in *Aspergillus fumigatus* and Other *Aspergillus* species. J. Clin. Microbiol..

[55] Monteiro C., Faria M.A., Pinheiro D., Lameiras C., Pinto E. (2018). First description of clinical *Aspergillus fumigatus cyp51A* TR_46_ /Y121F/T289A mutant in Portugal. J. Glob. Antimicrob. Resist..

[56] Sabino R., Veríssimo C., Viegas C., Viegas S., Brandão J., Alves-Correia M., Borrego L.-M., Clemons K.V., Stevens D.A., Richardson M. (2019). The role of occupational *Aspergillus* exposure in the development of diseases. Med. Mycol..

[57] Viegas C., Gomes A.Q., Faria T., Sabino R. (2015). Prevalence of *Aspergillus fumigatus* complex in waste sorting and incineration plants: An occupational threat. Int. J. Environ. Waste Manag..

[58] Viegas C., Faria T., Caetano L.A., Carolino E., Gomes A.Q., Viegas C. (2017). *Aspergillus* spp. prevalence in different Portuguese occupational environments: What is the real scenario in high load settings?. J. Occup. Environ. Hyg..

[59] Viegas C., Faria T., Monteiro A., Caetano L.A., Carolino E., Gomes A.Q., Viegas S. (2017). A Novel Multi-Approach Protocol for the Characterization of Occupational Exposure to Organic Dust—Swine Production Case Study. Toxics.

[60] Kano R., Kohata E., Tateishi A., Murayama S.Y., Hirose D., Shibata Y., Kosuge Y., Inoue H., Kamata H., Hasegawa A. (2014). Does farm fungicide use induce azole resistance in *Aspergillus fumigatus*?. Med. Mycol..

[61] O’Neill J. (2015). Antimicrobials in Agriculture and the Environment: Reducing Unnecessary Use and Waste. The Review on Antimicrobial Resistance. https://amr-review.org/sites/default/files/Antimicrobials%20in%20agriculture%20and%20the%20environment%20-%20Reducing%20unnecessary%20use%20and%20waste.pdf.

[62] Gisi U. (2014). Assessment of selection and resistance risk for demethylation inhibitor fungicides in *Aspergillus fumigatus* in agriculture and medicine: A critical review. Pest Manag. Sci..

[63] Chowdhary A., Kathuria S., Xu J., Sharma C., Sundar G., Singh P.K., Gaur S.N., Hagen F., Klaassen C.H., Meis J.F. (2012). Clonal Expansion and Emergence of Environmental Multiple-Triazole-Resistant *Aspergillus fumigatus* Strains Carrying the TR_34_/L98H Mutations in the *cyp51A* Gene in India. PLoS ONE.

[64] Verweij P.E., Chowdhary A., Melchers W.J.G., Meis J.F. (2016). Azole Resistance in *Aspergillus fumigatus*: Can We Retain the Clinical Use of Mold-Active Antifungal Azoles?. Clin. Infect. Dis..

[65] Ashu E.E., Hagen F., Chowdhary A., Meis J.F., Xu J. (2017). Global Population Genetic Analysis of *Aspergillus fumigatus*. MSphere.

